# Identification of functional signatures in the metabolism of the three cellular domains of life

**DOI:** 10.1371/journal.pone.0217083

**Published:** 2019-05-28

**Authors:** Pedro Escobar-Turriza, Rafael Hernandez-Guerrero, Augusto Cesar Poot-Hernández, Katya Rodríguez-Vázquez, Jorge Ramírez-Prado, Ernesto Pérez-Rueda

**Affiliations:** 1 Instituto de Investigaciones en Matemáticas Aplicadas y en Sistemas, Universidad Nacional Autónoma de México, Unidad Académica Yucatán, Mérida, Yucatán, México; 2 Centro de Investigación Científica de Yucatán, Col. Chuburná de Hidalgo, Mérida, Yucatán, México; 3 Departamento de Ingeniería de Sistemas Computacionales y Automatización, Instituto de Investigaciones en Matemáticas Aplicadas y en Sistemas, Ciudad Universitaria, Universidad Nacional Autónoma de México, Ciudad de México, México; 4 Instituto de Fisiologia Celular, Ciudad Universitaria, Universidad Nacional Autónoma de México, Ciudad de México, México; 5 Centro de Genómica y Bioinformática, Facultad de Ciencias, Universidad Mayor, Santiago, Chile; Griffith University, AUSTRALIA

## Abstract

In order to identify common and specific enzymatic activities associated with the metabolism of the three cellular domains of life, the conservation and variations between the enzyme contents of *Bacteria*, *Archaea*, and *Eukarya* organisms were evaluated. To this end, the content of enzymes belonging to a particular pathway and their abundance and distribution in 1507 organisms that have been annotated and deposited in the KEGG database were assessed. In addition, we evaluated the consecutive enzymatic reaction pairs obtained from metabolic pathway reactions and transformed into sequences of enzymatic reactions, with catalytic activities encoded in the Enzyme Commission numbers, which are linked by a substrate. Both analyses are complementary: the first considers individual reactions associated with each organism and metabolic map, and the second evaluates the functional associations between pairs of consecutive reactions. From these comparisons, we found a set of five enzymatic reactions that were widely distributed in all the organisms and considered here as universal to *Bacteria*, *Archaea*, and *Eukarya*; whereas 132 pairs out of 3151 reactions were identified as significant, only 5 of them were found to be widely distributed in all the taxonomic divisions. However, these universal reactions are not widely distributed along the metabolic maps, suggesting their dispensability to all metabolic processes. Finally, we found that universal reactions are also associated with ancestral domains, such as those related to phosphorus-containing groups with a phosphate group as acceptor or those related to the ribulose-phosphate binding barrel, triosephosphate isomerase, and D-ribose-5-phosphate isomerase (RpiA) lid domain, among others. Therefore, we consider that this analysis provides clues about the functional constraints associated with the repertoire of enzymatic functions per organism.

## Introduction

In recent years, the organization and construction of metabolic databases, such as KEGG [1] and MetaCyc [2], has allowed the understanding of adaptive process of cellular life, the diversity of cellular organization, and the complexity of the cellular systems [3]. Metabolism is considered a biological network, where enzymes or substrates are represented as nodes and edges represent their relationships [4–6]. In this context, two possible scenarios have been suggested to explain the emergence and evolution of metabolic pathways, based on the fact that gene duplication, followed by divergence, lead to the origin of new metabolic reactions. The *Stepwise* scenario [7] suggests that when a substrate tends to be depleted, gene duplication provides an enzyme capable of supplying the absent substrate, giving rise to homologous enzymes that catalyze consecutive reactions. In contrast, the *Patchwork* scenario [8] proposes that duplication of genes encoding promiscuous enzymes (capable of catalyzing multiple reactions) allows each descendant enzyme to specialize in one of the ancestral reactions. Based on these hypotheses, it is plausible that a small number of enzymes with broad specificity existed in early stages of metabolic evolution. Genes encoding these enzymes would have been duplicated, generating enzymes that, through sequence divergence, became more specialized [9].

Comparative analysis of metabolism provides insights into the identification of enzyme recruitment and duplication events. In this regard, it has previously described that metabolic pathways exhibit high retention of duplicated enzymes within functional modules and coupling of biochemical reactions [10–13]. In this work, we evaluated how the individual and consecutive pairs of enzymatic reactions (by using the Enzymatic Commission (EC) numbers) are distributed along the metabolism of the *Bacteria*, *Archaea*, and *Eukarya* cellular domains and how this distribution has influenced the metabolic pathways in their actual form. To this end, the information of metabolic maps of 1507 organisms that have been deposited in the KEGG database were evaluated in terms of their enzymatic composition. In addition, the functional conservation between consecutive pairs of enzymatic reactions were analyzed considering the metabolic pathways as linear enzymatic step sequences. Finally, the contents of structural domains were evaluated in terms of their Superfamily database assignments, which allowed us to identify universal reactions that are also associated with ancestral domains, such as those related to phosphorus-containing groups with a phosphate group as acceptor or those related to the ribulose-phosphate binding barrel, triosephosphate isomerase, and D-ribose-5-phosphate isomerase (RpiA) lid domain, among others. Therefore, we consider that this analysis provides clues about the functional constraints associated with the repertoire of enzymatic functions per genome.

## Materials and methods

### Distribution of enzymatic reactions

In order to determine the distribution of enzymatic reactions in all the organisms, 195 different EC numbers, considering the first three classification levels, were traced along 105 archaeal, 1264 bacterial, and 138 eukaryal genomes. The rates of occurrence of each EC number per organism and per taxonomical division were calculated, considering the presence (a value of 1) and absence (value of 0) of the enzyme (EC number), using the following formula:

$$RA=\frac{Ni}{ODiv}$$

Where, *RA* = relative abundance of EC numbers;

*i = 1… n* taxonomical divisions;

*N* = total occurrence of each EC number per taxonomical division; and

*ODiv* = total number of organisms per taxonomical division.

Fifty taxonomic divisions (according to the NCBI classification system) were considered: among *Bacteria* were the divisions *Acidobacteria*, *Actinobacteria*, *Alphaproteobacteria*, *Aquificae*, *Bacteroidetes*, *Betaproteobacteria*, *Deltaproteobacteria*, *Gammaproteobacteria*, *Epsilonproteobacteria*, Other *Proteobacteria*, *Chlamydiae*, *Chlorobi*, *Chloroflexi*, *Chrysiogenetes*, *Cyanobacteria*, *Deferribacteres*, *Deinococcus-Thermus*, *Dictyoglomi*, *Elusimicrobia*, *Fibrobacteres*, *Firmicutes-Bacilli*, *Firmicutes-Clostridia*, *Firmicutes-*Others, *Fusobacteria*, *Gemmatimonadetes*, *Nitrospirae*, *Planctomycetes*, *Spirochaetes*, *Synergistetes*, *Tenericutes*, *Thermotogae*, Unclassified *Terrabacteria* group, and *Verrucomicrobia*. Among *Archaea* were the divisions *Crenarchaeota*, *Euryarchaeota*, *Korarchaeota*, *Nanoarchaeota*, and *Thaumarchaeota*. The *Eukarya* divisions included *Alveolata*, *Amoebozoa*, *Choanoflagellida*, *Diplomonadida*, *Euglenozoa*, Fungi, *Heterolobosea*, *Metazoa*, *Parabasalia*, *Rhodophyta*, *Stramenopiles*, and *Viridiplantae*. (See S1 Table for a complete description of all organisms considered in this analysis). Finally, the RA represented in a matrix showing the presence of each EC number per division was analyzed with a Hierarchical Clustering Approach (HCA) using a complete linkage algorithm, with the Pearson correlation as a similarity measure, as implemented in the Mev4 program [14].

### Construction of enzymatic step sequences

A total of 144 metabolic maps associated with 1507 genomes of Bacteria, Archaea and Eukarya were downloaded from the KEGG database and stored in KGML files, v. 0.71. S2 Table. Metabolic pathways were transformed into 1,420,221 linear Enzymatic Step Sequences (ESS) by using the Breadth-First search algorithm [15], which gathers the closer neighbor for each enzyme by considering the substrate/product linked them, as it has reported [16, 17]. In this regard, the first three levels of EC numbers were considered to represent an enzyme as a string or a set of consecutive enzymatic steps, as it was previously suggested [16, 17]. In order to eliminate redundancy associated to the ESS, two filters were applied: a) if two ESS from different organisms but the same metabolic maps were identical, then only one of them was considered, leaving a set of 57,095 total ESS representative or nonredundant ESS (nrESS); and b) if two identical sequences from the same metabolic map and organism had different lengths, the longer sequence was only considered, leaving a set of 27,991 nrESS. From these nrESS, 3151 possible consecutive pairs of enzymatic reactions were obtained, and their distributions across all the genomes were evaluated in a similar way as individual EC numbers.

### Distribution of EC numbers per metabolic map

The distribution of individual and pairs of enzymatic reactions were traced across the 144 metabolic maps deposited in the KEGG database. A matrix showing the presence and absence of EC numbers and pairs of enzymatic reactions was constructed, and the relative distribution was calculated. The rate of occurrence of each EC number per metabolic map was calculated, based on its presence (a value of 1) or absence (value of 0).

### Domain assignment of EC number

Each enzymatic reaction was associated with its respective protein in the genome, and its protein domain structure organization was determined using the Superfamily database version 1.75. For this, 1507 complete genomes were scanned against a library of 1659 superfamily HMM models in the HMMer program version 3.1b2 [18], with an E-value of ≤10^−3^.

## Results and discussion

### Abundance of enzymatic reactions across all the cellular domains

In order to evaluate the abundance of enzymatic reactions, the metabolism information for 1264 *Bacteria*, 105 *Archaea*, and 138 *Eukarya* organisms was downloaded from the KEGG database and exhaustively scrutinized. The EC numbers represented by the first three levels were obtained from the metabolic maps deposited in the KEGG database, as it has been previously suggested [16, 17]. From these enzymatic reactions, 43.87% are annotated as transferases (EC:2), 21.93% as oxidoreductases (EC:1), 17.22% as lyases (EC:4), 13.44% as hydrolases (EC:3), 12.75% as ligases (EC:6), 8.32% as isomerases (EC:5), and 0.10% as translocases (EC:7). This distribution suggests that enzyme-catalyzed transfer and oxidoreduction reactions are highly abundant in metabolism, probably because metabolic processes can be seen as the movement of electrons between molecules, often capturing some of the energy released as the electrons move from high-energy to lower-energy states, as occurs in glycolysis or respiration [15].

**Fig 1:**
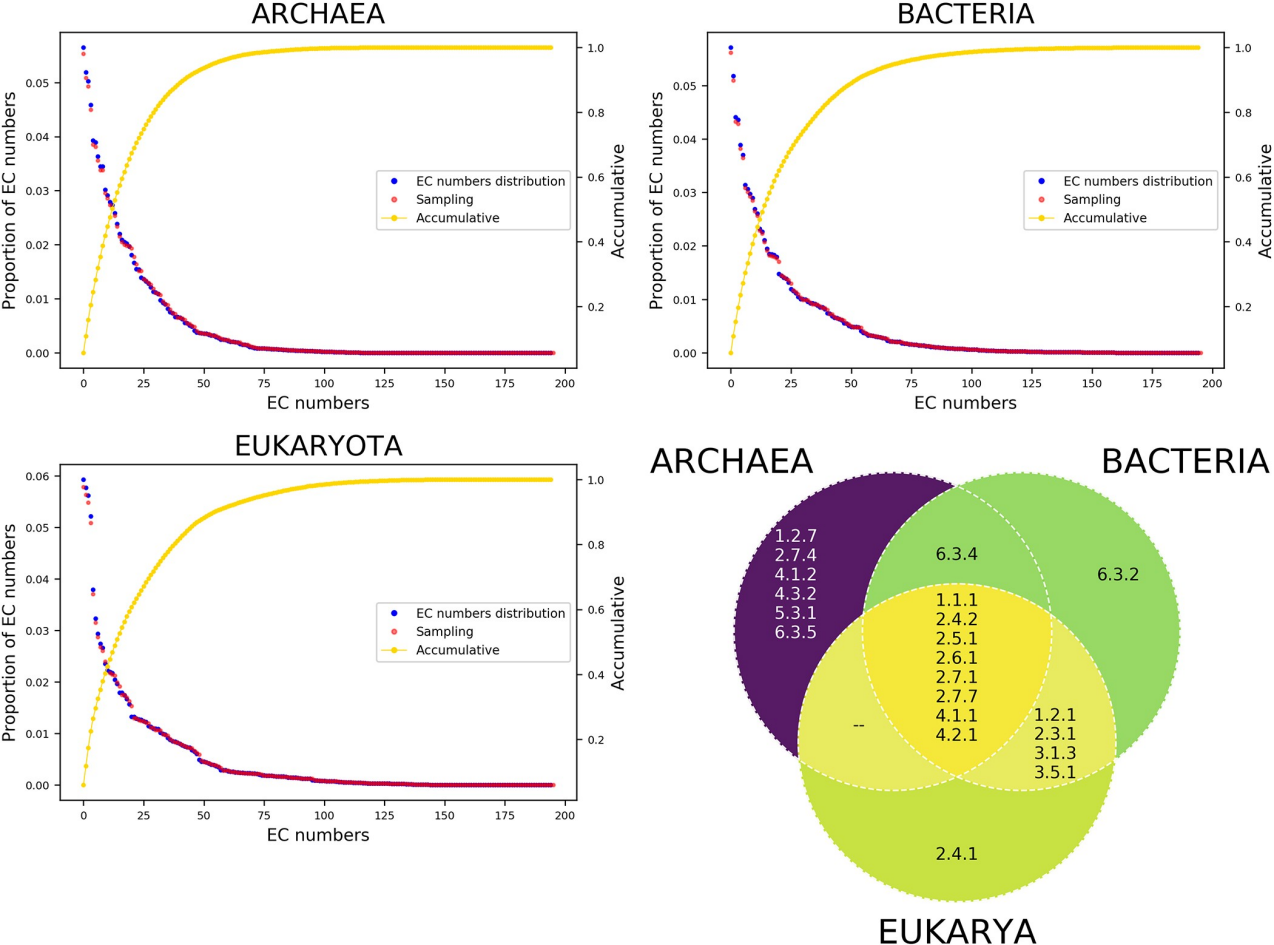
Abundance of EC numbers. a) *Archaea*, b) *Bacteria*, and c) *Eukarya*. The Y-axes indicate the proportion of EC numbers per cellular domain; the X-axes indicate the EC numbers. Each data point corresponds to one EC number. The intersection with the yellow plot indicates the top of the most abundant EC numbers and their percentages. The secondary axes indicate the cumulative proportion of EC numbers. d) The Venn diagram (lower right) shows common and specific EC numbers identified in the three cellular domains.

To determine the abundance of specific enzymatic reactions, the 195 different EC numbers (considering the three levels of information) were traced across all genomes divided into their respective cellular domains. To this end, upper values at the intersection between a relative and a cumulative relative frequency were considered a threshold of most abundant EC reactions. From this, we found highly abundant EC numbers were associated with the analyzed organisms, such as in *Archaea*, where 15 EC numbers represent 55.2% of the total of EC numbers (Fig 1A). In contrast, 14 EC numbers represent 49.2% of *Bacteria*, and 13 EC numbers represents 44.6% of the total of EC numbers of *Eukarya* (Fig 1B and 1C**).** From these abundance levels, eight enzymatic activities (EC 1.1.1, 2.4.2, 2.5.1, 2.6.1, 2.7.1, 2.7.7, 4.1.1, and 4.2.1) were identified as highly abundant in the three cellular domains; these groups are mainly devoted to transferases (Fig 1D). One EC number was identified as abundant in *Archaea* and *Bacteria* (6.3.4) but not in *Eukarya*; four EC numbers (1.2.1, 2.3.1, 3.1.3, and 3.5.1) are abundant in *Bacteria* and *Eukarya* but not in *Archaea*; one ligase (6.3.2) was identified as abundant in *Bacteria* but not in *Archaea* and *Eukarya*; one EC number (2.4.1) was abundant in *Eukarya*; finally, six activities (1.2.7, 2.7.4, 4.1.2, 4.3.2, 5.3.1, and 6.3.5) were identified as highly abundant only in *Archaea* (Fig 1A).

In order to exclude a bias as a consequence of overrepresentation of bacterial, archaeal, or eukaryotic genomes in the observed results, we performed an analysis considering random subsets of organisms for the three cellular domains. In the process, we randomly selected 100 genomes per domain 1000 times, obtained the average of each one, and compared the result against the original distribution (considering the complete set of genomes). From these analyses, we found consistency between sampling and observed data, suggesting that our results are sufficiently robust and confirming that 15 enzymatic activities are abundant in *Archaea*, 14 in *Bacteria*, and 13 in *Eukarya*, as we also found when we considered the complete dataset.

One of the most recurrent enzymatic activities identified in all the organisms corresponded to transferases of phosphorus-containing groups (2.7), in particular, the nucleotidyl phosphotransferases (2.7.7) involved in the transfer of acyl, glycosyl, amino, and phosphate (includes diphosphate, nucleotidyl residues, and others). In contrast, the phosphotransferases (2.7.4) were abundant in *Archaea*; such enzymes are involved in the addition of phosphate to UMP and CMP molecules, among other molecules. Accordingly, based on network simulations, transferase activities were found to be associated with new metabolic pathways, in particular, with multifunctional enzymes as a consequence of dependence toward the donator or acceptor metabolite [19, 20].

In summary, we identified eight enzymatic reactions as the most abundant activities in all the organisms analyzed in this work, suggesting a recurrent set of functions used in all the organisms, that shaped the of metabolic pathways in all the organisms (Fig 1D).

### Distribution of EC numbers among all organisms

**Fig 2:**
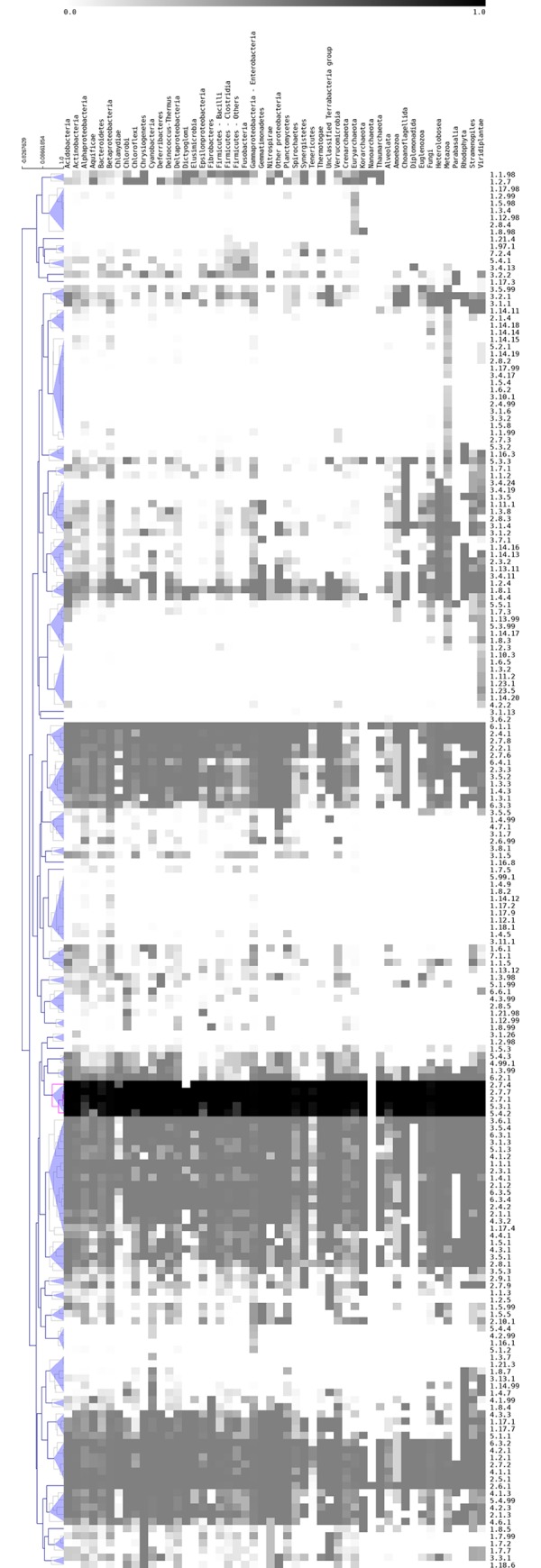
Clustering of EC numbers displaying the presence of a cluster of enzymatic activities in all organisms. A total of 195 EC numbers were grouped with an HCA using the Pearson squared correlation as the distance metric. Fifty clusters were obtained using a distance threshold of 0.668. A cluster of five EC numbers (2.7.4, 2.7.7, 2.7.1, 5.3.1, and 5.4.2) are widely distributed in 50 taxonomic divisions, with an RA of ≥ 0.95 (pink branches and highlighted in black).

A natural question is whether the most abundant EC numbers are also the most widely distributed in all the organisms, i.e., they are ubiquitous. Therefore, the distribution of EC numbers in the three cellular domains was determined and an HCA was achieved. Based on this distribution, five enzymatic reactions (three transferases, 2.7.4, 2.7.7, and 2.7.1; two isomerases, 5.3.1 and 5.4.2) were clustered together and found to be widely distributed among all organisms, with a relative abundance of ≥ 0.95 (Fig 2), suggesting an ancestral catalytic activity (Table 1). From these, the EC 2.7.7 and 2.7.1 reactions were identified as highly abundant in all the organisms, as previously described, whereas EC 2.7.4 was identified as abundant in *Archaea*. The two isomerase catalytic reactions, corresponding to ECs 5.3.1 and 5.4.2, were not identified as abundant, suggesting that ubiquitous catalytic reactions are not necessarily abundant in all the organisms.

**Table 1:** Five EC numbers are universally distributed in Bacteria, Archaea and Eukarya. In the fourth column is indicated the frequency of each protein domain identified by Superfamily searches.

EC number	Description	Function	Total number of structural domains	Most abundant structural domains (ID Superfamily/Description/%)
**2.7.1**	Transferase	Phosphotransferase with alcohol group as acceptor	261	53067 / Actin-like ATPase domain/ 0.183274 52540 / P-loop-containing nucleoside triphosphate hydrolases/ 0.152635 54211 / Ribosomal protein S5 domain 2-like/ 0.064262
**2.7.4**	Transferase	Phosphotransferase with phosphate group as acceptor	48	52540 / P-loop-containing nucleoside triphosphate hydrolases / 0.375094
**2.7.7**	Transferase	Nucleotidyl transferase	692	53448 / Nucleotide-diphospho-sugar transferases / 0.077639 52540 / P-loop-containing nucleoside triphosphate hydrolases / 0.052910 52374 / Nucleotidyl transferase / 0.051996 55979 / DNA clamp / 0.050308 81301 / Nucleotidyl transferase/ 0.042375 54211 / Ribosomal protein S5 domain 2-like / 0.039876 55666 / Ribonuclease PH domain 2-like/ 0.039296 56672 / DNA/RNA polymerases/ 0.037970
**5.3.1**	Isomerase	Interconverting aldoses and ketoses	326	51366 / Ribulose-phosphate binding barrel / 0.271909 51351 / Triosephosphate isomerase / 0.108794 53697 / SIS domain / 0.106178 51182 / RmlC-like cupins / 0.065631 51395 / FMN-linked oxidoreductases / 0.063015 100950 / NagB/RpiA/CoA transferase-like / 0.059398
**5.4.2**	Isomerase	Phosphotransferase (phosphomutase)	12	53738 / Phosphoglucomutase, first 3 domains / 0.400197 53254 / Ph osphoglycerate mutase-like / 0.215499

Thus, we asked whether these universal EC numbers are also widely distributed among all metabolic pathways. To this end, all the EC numbers were located on the 144 maps, emphasizing the universal reactions, and they were identified them in 37 of 144 metabolic maps, mainly those associated with amino acid metabolism (histidine and glycine); carbohydrate metabolism, such as galactose and fructose metabolism; cofactor (riboflavin and thiamine) metabolism; and nucleotide metabolism. Almost all of these ubiquitous reactions were also associated with ancient metabolic pathways such as histidine metabolic pathway that has been proposed as present in the last common ancestor of all the organisms or the inosine 5’-monophosphate (IMP) in purine metabolism that could be involved in thiamine synthesis or its derivatives in early stages of cellular evolution [10, 12].

In counterpart, nonuniversal EC numbers were found in low proportions in diverse cellular divisions, such as *Chlamydia* and *Tenericutes* (*Bacteria*), *Nanoarchaeum* (*Archaea*), and *Parabasalia* and *Diplomonadida* (*Eukarya*). This decrease in the proportion of enzymatic reactions correlated with the few metabolic maps associated with them, probably because these organisms are associated with specific and constrained environments, such as *Nanoarchaeum equitans*, an endosymbiont of *Ignococcus* sp.

### How ancient are the protein domains associated with the EC numbers?

To determine if the universal and specific catalytic reactions previously identified are associated with ancestral protein domains, all EC numbers were evaluated in terms of their domain organization based on Superfamily database assignments [21]. This information is relevant under the hypothesis that the most abundant and ubiquitously distributed EC numbers, as previously described, are associated with ancient protein domains. To evaluate this hypothesis, protein domains identified by Superfamily assignments were traced along the ancestry according to the approach described by Caetano-Anolles et al. [19]. In brief, the approach considers the timeline of enzyme evolution spanning ~3.8 billion years of evolution, where “0” represents the origin of enzymes and “1” represents present day. Therefore, the ancestrality is defined by ancestries of protein domain constituents derived from a structural phylogenomic census [22].

**Fig 3:**
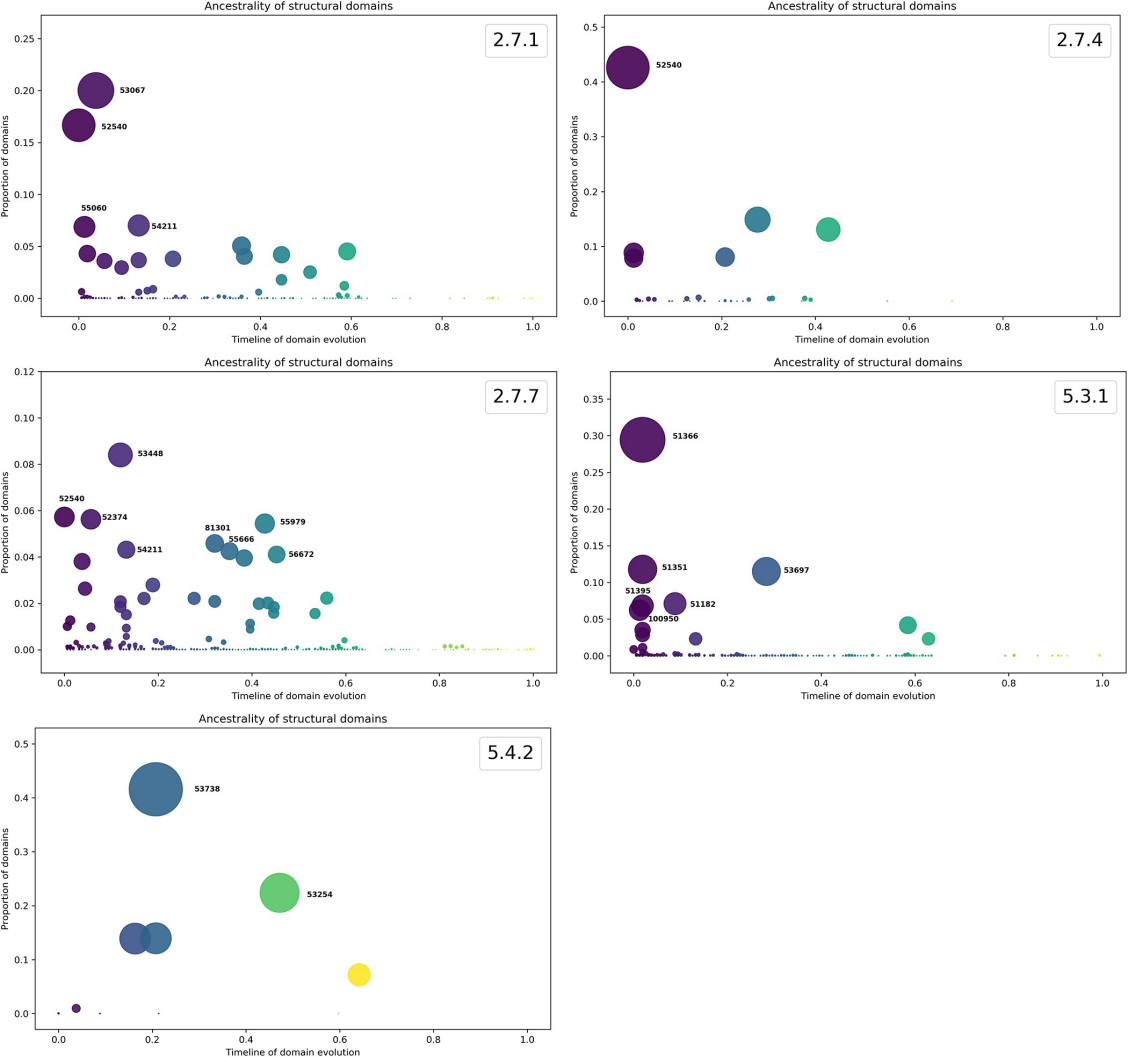
**Ancestrality and abundance of the structural domains of universal enzymatic reactions: a) 2.7.1, b) 2.7.4, c) 2.7.7, d) 5.3.1 and e) 5.4.2.** The timeline assigns how ancient each structural domain present in the universal enzymatic reactions is, as suggested by Caetano-Anollés et al [19].

The EC 2.7.1 catalytic enzyme transfers phosphorus-containing groups with an alcohol group as acceptor. Associated to proteins with this enzymatic function, 261 different structural domains were identified, and these were mainly devoted to phosphate activities, such as the Actin-like ATPase domain, PTS system IIB component-like; Ribokinase-like, and GHMP Kinase C-terminal domain, among others. Thus, this enzymatic activity preferentially uses the Actin-like ATPase domain (SF:53067), P-loop-containing nucleoside triphosphate hydrolases (SF:52540), and Ribosomal protein S5 domain 2-like (SF:54211), since they represent 40% of the repertoire of domains (Fig 3A and Table 1). The high diversity of protein domains associated with this activity suggests multiple recruitment events of protein domains along the history of life, indicating that recruitment of catalytic functions is quite important for increasing the size of metabolic maps and to maintain integrity of metabolic functions.

The second group of enzymatic activity corresponds to phosphotransferases with a phosphate group as acceptor (EC 2.7.4). Proteins that carry on this activity have been related to 48 different domains, mainly devoted to phosphate activities, such as the Carbamate kinase-like; Phospholipase D-nuclease; Ribokinase-like, and Nucleoside diphosphate kinase NDK, among others. Fig 3B. In this regard, the most abundant domain associated with this catalytic activity is related to P-loop-containing nucleoside triphosphate hydrolase (SF:52540), which is considered as the most ancient fold architecture and closer to the last common ancestor of all organisms, considering a phylogenomic reconstruction of evolutionary relationships at fold level [3, 23, 24] representing 37% of the total number of protein domains identified in this activity. Indeed, Alva et al [25] compare domains representative of known folds and identified the P-loop as one of the 40 fragments whose similarity and function suggest a primordial role closer to the RNA-world. Posterior domain recruitment events could be also shaped the EC 2.7.4 enzymatic function.

Proteins that carry on nucleotidyl transferase activity (EC 2.7.7) have been related to 692 different domains, mainly devoted to phosphate activities, such as the Nucleotide-diphospho-sugar transferases, Nucleotidylyl transferase and Nucleotidyl transferases, among others. Eight domains represent 39.2% of the total set of domains identified with this enzymatic activity, with Nucleotide-diphospho-sugar transferases (SF:53448) being the most abundant domain associated with this catalytic activity, followed by the ancient P-loop-containing nucleoside triphosphate hydrolase (SF:52540) [3] (Fig 3C and Table 1). It seems that multiple recruitment events along the timeline of domain evolution have shaped the nucleotidyl transferase activity (EC 2.7.7) in all the organisms.

The isomerases that interconvert aldoses and ketoses (EC 5.3.1) are proteins related to 326 different domains, mainly devoted to phosphate activities, such as the Ribulose-phosphate binding barrel, Triosephosphate isomerase, and D-ribose-5-phosphate isomerase (RpiA) lid domain, among others. Fig 3D. Among these, six domains represent 67% of the total of 326 domains. Indeed, the Ribulose-phosphate binding barrel is the most abundant domain associated with this activity, and it is also considered one of the most ancient domains (Table 1).

Finally, the Phosphotransferases (phosphomutase; EC 5.4.2) are associated with 12 different domains, mainly devoted to phosphate activities, such as the Ribonuclease H-like or the P-loop-containing nucleoside triphosphate hydrolases. Fig 3E. Only two domains account for 61.5% of the total protein domains, the most abundant being Phosphoglucomutase (SF:53738), followed by the Phosphoglycerate mutase-like (SF:53254) (Table 1).

In summary, the five enzymatic functions identified as ubiquitous were related to domains associated with phosphate-related functions (transferases and isomerases), supporting the importance of phosphorus metabolism in the global maintenance of cellular function. Ancestral functions identified in all the organisms, such as those enzymatic activities associated with the domain P-loop-containing nucleoside triphosphate hydrolases (SF:52540), are the most recurrent and most ancestral among the universal enzymes (Fig 3). In contrast, among the set of nonuniversal EC numbers, the most abundant domain corresponds to the ancient NAD(P)-binding Rossmann fold domain [26], which is associated with fundamental functional processes, such as FAD, NAD, or NADP binding [27, 28].

### Functional relationships of all EC consecutive pairs show conserved and variable taxonomic groups

To evaluate the functional associations between all the EC numbers, we analyzed the distribution of all nonredundant EC consecutive pairs in a database of nrESS. We consider that this analysis is important to determine how EC numbers are functionally linked to other EC numbers. To determine the most significant EC pairs, frequencies of consecutive reaction types (EC:a.b.c → EC:w.x.y) were compared against the expected values, using a set of random ESS. In this regard, 10 random databases were constructed by shuffling the real nrESS, maintaining the EC composition and lengths, as similar to the random ESS constructed for Proteobacteria comparisons [16, 17]. Therefore, the random databases were used to extract the consecutive reaction pairs, and a Z-score was calculated [Z-score (Z_*i*_) = (Nreal i—<Nrandi>) / std (Nrandi)]. From this, a Z-score of ≥5 suggests that the frequency of the pair in the real ESS is significantly greater than expected by chance, leaving a set of 132 EC pairs as significant, suggesting that they are involved in a large number of consecutive reactions in the organisms considered in this analysis.

Based on the distribution pattern associated with the enzymatic pairs in all the genomes, we identified five pairs (ECs 4.2.1:5.4.2; 5.4.2:4.2.1; 2.7.7:2.7.1; 2.7.4:3.6.1; and 2.7.7:2.7.8) as widely distributed among the organisms or “universal” enzymatic pairs. (Table 2). These reactions are mainly involved in phosphate-related functions (transferases and isomerases) and also related to phosphorus metabolism.

**Table 2:** EC pairs universally distributed. In the fourth column is the frequency of each protein domain, identified by Superfamily searches.

*EC number pair (A*:*B)*	*Description*	*Function EC A*	*Function EC B*
**4.2.1:5.4.2**	Lyase:Isomerase	Hydrolyase	Phosphotransferase (phosphomutase)
**5.4.2:4.2.1**	Isomerase:Lyase	Phosphotransferase (phosphomutase)	Hydrolyase
**2.7.7:2.7.1**	Transferase:Transferase	Nucleotidyl transferase	Phosphotransferase with alcohol group as acceptor
**2.7.4:3.6.1**	Transferase:Hydrolase	Phosphotransferase with phosphate group as acceptor	In phosphorus-containing anhydrides
**2.7.7:2.7.8**	Transferase:Transferase	Phosphotransferase (phosphomutase)	Transferase for other substituted phosphate groups

Finally, to evaluate the roles of these enzymatic pairs in all the metabolic maps, these “universal” reactions were traced along the complete metabolism of *Bacteria*, *Archaea*, and *Eukarya*. Therefore, the five reactions were identified in the glycerolipid metabolism, probably because this pathway is a fundamental pathway associated with the origin and evolution of cell membranes and linked to the central structural component of the major classes of biological lipids, triglycerides, and phosphatidyl phospholipids, which are involved in the composition of membranes [29]. In this regard, diverse lipid structures have been identified in the three cellular domains, such as the ester bond in long chain fatty acids in Bacteria and Eukarya or ether lipids with isoprenoids in Archaea; there is a common polar lipids with a glycerol backbone in all the organisms, with the exception of their stereostructures [30]. Therefore, this common backbone associated to the organisms analyzed in this work, however further analyses are required.

Finally, two ancient metabolic maps, for glycolysis and methane, contain two and three reaction pairs, respectively, 5.4.2:4.2.1 and 4.2.1:5.4.2 and 5.4.2:4.2.1, 4.2.1:5.4.2, and 2.7.7:2.7.8; whereas the 2.7.7:2.7.1 pair is preferentially associated with eight metabolic maps, outstanding among which are the amino sugar and nucleotide sugar metabolism, fructose and mannose metabolism maps, among others.

## Conclusions

Enzymatic activities reflect the organization of metabolism in all organisms, and their analysis can provide clues about how reactions have shaped to their actual form. In this regard, we evaluated the abundance and distribution of enzymatic reactions in organisms from three cellular domains, and we found five EC numbers (at the first three levels of EC classification) were universal to *Bacteria*, *Archaea*, and *Eukarya*, although they are constrained to specific metabolic maps (i.e., they are not associated with all metabolic maps). In addition, we identified that those reactions are associated with ancient folds, as the P-loop-containing nucleoside triphosphate hydrolase (SF:52540), suggesting that universal reactions could be also ancestral in the evolution of metabolic pathways. When we analyzed the functional association between the enzymatic reactions, 132 EC pairs of reactions were identified as significant, and only 5 of them were identified as universal to the cellular domains. In summary, we found that conserved enzymatic reactions are mainly related to phosphorylation reactions, which are an essential on the modern metabolism.

## Supporting information

S1 Table Full description of 1507 organisms based on the NCBI classification system. Columns correspond to: 1. Name of organism at NCBI database; 2. Organism ID in KEGG database; 3. Total number of proteins by organism; 4. Total number of non redundant ESS by organism; 5. Total of non-redundant EC numbers; 6. Total of maps by organism; 7. Taxonomy according of NCBI classification system; 8. Organism lifestyle; and 9. Other features of lifestyles
(TXT)

S2 Table Full description of metabolic maps. Columns correspond to: 1. Metabolic map ID in KEGG database; 2. Metabolic map name; 3. Total number of reactions; 4. Total number of non-redundant reactions; 5. Total number of ESS by metabolic map; and 6. Total number of non-redundant ESS by metabolic map.
(TXT)
